# Impact of Acute Blood Loss on Clinical, Hematological, Biochemical, and Oxidative Stress Variables in Sheep

**DOI:** 10.3390/vetsci9050229

**Published:** 2022-05-11

**Authors:** Rejane Santos Sousa, Caroline Santos Sousa, Francisco Leonardo Costa Oliveira, Paulo Ricardo Firmino, Isadora Karolina Freitas Sousa, Valeria Veras Paula, Nohora Mercado Caruso, Enrico Lippi Ortolani, Antonio Humberto Hamad Minervino, Raimundo Alves Barrêto-Júnior

**Affiliations:** 1 School of Veterinary Medicine and Animal Science, University of São Paulo, Av. Prof. Orlando Marques de Paiva 87, Cidade Universitária, São Paulo 05508-270, SP, Brazil; rejane.sousa@unifesspa.edu.br (R.S.S.); carolinesantos@unifesspa.edu.br (C.S.S.); oliveiraflc@usp.br (F.L.C.O.); isadora.sousa@ifam.edu.br (I.K.F.S.); ortolani@usp.br (E.L.O.); 2 Department of Animal Science, Federal Rural University of the Semiarid Region, Av. Francisco Mota 572, Bairro Costa e Silva, Mossoró 59625-000, RN, Brazil; pauloricardo83@hotmail.com (P.R.F.); valeria@ufersa.edu.br (V.V.P.); barreto@ufersa.edu.br (R.A.B.-J.); 3 Departamento Productividad e Innovación, Universidad de la Costa (CUC), Calle 58 n.55-66, Barranquilla 080001, Colombia; nmercado1@cuc.edu.co; 4 Laboratory of Animal Health, LARSANA, Federal University of Western Pará (UFOPA), Rua Vera Paz S/N, Salé, Santarém 68040-255, PA, Brazil

**Keywords:** anemia, ovine, superoxide dismutase, glutathione peroxidase, catalase

## Abstract

Blood loss in sheep can have different causes and may result in anemia. We aimed to evaluate the clinical, hematological, and biochemical alterations and the oxidative stress generated by acute blood loss. Eighteen healthy sheep underwent phlebotomy to remove 40% of the blood volume and were evaluated clinically and by laboratory tests for clinical, biochemical, and blood gas variables and to assess oxidative stress before induction (T0), 30 min (T30 min), and 6 (T6 h), 12 (T12 h), and 24 h (T24 h) after blood loss. The sheep showed tachycardia from T30 min until T24 h, reduction in the hematocrit, number of erythrocytes, and hemoglobin concentration, with lower values at T24 h and increase in the number of leukocytes from T12 h on. There was a reduction in blood pH and oxygen pressure at T30 min, increased lactate concentration and reduced blood bicarbonate at this time. There was an increase in urea concentration from T6 h until the end of the study, with no change in creatinine levels. The animals did not show changes in the concentration of malonaldehyde, and in the activity of the enzymes superoxide dismutase, glutathione peroxidase, and catalase, but there was a reduction in the concentration of reduced glutathione at T24 h. The acute loss of 40% of blood volume is capable of promoting relevant clinical, hematological, blood gas, and biochemical alterations, and contributed to the appearance of oxidative stress with reduced glutathione concentration, suggesting that this process generated free radicals in sufficient quantity to diminish the action of antioxidants.

## 1. Introduction

Sheep farming in Brazil presented an interesting pattern in the last decades, which include a large decrease in sheep herds, with a reduction of almost 30% from 1990 to 2002, mostly due to reduction of wool production, followed by a return of grow beginning at 2002 driven by meat production [1,2,3]. By 2020, the total sheep heard in Brazil was higher than 20.5 million heads, with 70% of those located in the northeast region [4].

Sheep flocks can be affected by acute or chronic blood loss, a condition that can have different causes, such as trauma, post-partum surgeries and hemorrhages, and parasitic diseases [5]. The acute loss of blood can generate anemia, which is characterized by a reduction in the body’s ability to supply the tissues with the appropriate amount of oxygen, in addition to changes in homeostasis, which can lead to hypovolemic shock and even death of the animal [6,7,8], requiring in most severe cases blood transfusion [9].

In addition to the hematological, blood gas, and biochemical changes caused by acute blood loss, oxidative stress may also occur; however, the mechanism by which anemia contributes to oxidative stress is not fully elucidated. It is believed that inadequate tissue oxygenation is accompanied by increased production of free radicals determining changes in cellular energy metabolism, increased catecholamine catabolism, and leukocyte activation [10,11]. Oxidative stress develops when there is an imbalance between reactive oxygen species (ROS) production and removal. Excess ROS can promote lesions in cell membranes due to lipid peroxidation, protein oxidation, DNA modification/breakdown, and depolymerization of polysaccharides [11,12]. Research shows that chronic blood loss promotes oxidative stress mainly due to iron deficiency generated by parasitism or chronic inflammatory disease in sheep [13], as well as hemolysis due to parasitism by Babesia ovis [14], but the effect of acute blood loss on the generation of free radicals, which can damage the red blood cell membrane and reduce its half-life, is not known. A study with transfusion of stored blood in sheep [15] showed a reduction in the activity of the enzyme reduced glutathione after acute blood loss and this variable returned to normal values after blood transfusion, suggesting that the generation of free radicals may be reduced in cases of acute anemia when red blood cells are responsive, which can reduce oxidative damage to red blood cells.

Although research focusing on animal blood therapy has received some attention in recent years, with research available about blood preservation and transfusion in a wide range of animal species [15,16,17,18,19], the basic understanding of physiological alteration occurring after acute blood loss are still limited, especially in ruminants. Thus, this study aims to evaluate the clinical, hematological, biochemical, and oxidative stress changes in sheep subjected to acute blood loss.

## 2. Materials and Methods

The study was approved by the Ethics Committee on Animal Use (CEUA) of the Federal Rural University of the Semi-Arid (UFERSA), under protocol No. 23091.004731/2011-19.

### 2.1. Animals and Feeding

Eighteen healthy, three- to four-year-old, non-pregnant Santa Inês crossbred sheep, weighing on an average 52.89 ± 6.7 kg, were used. The selected animals remained in collective stalls and underwent a 30-day adaptation period. During this period, the animals received endectocide (Cydectin, Zoetis, São Paulo, Brazil), clostridial vaccine, and a broad spectrum coccidiostatic drug (Coccifin, Ouro Fino Animal Heatlh, Cravinhos, Brazil) for three consecutive days. All drugs were administered according to manufacturer recommendations.

The animals were fed a basal diet that surpass maintenance requirements [20], calculated at 2.3% of live weight, composed of 75% dry matter of *Cynodon dactylon* (89.2% dry matter, 31.2% crude fiber, 8.5% crude protein, 1.8% ether extract, and 6.4% minerals) grass hay and 25% commercial concentrate feed (88% dray mater, 10% crude fiber, 18% crude protein, 2.5 ether extract, and 10% minerals), which was provided in fractions twice a day. The sheep were supplemented with micro mineral mixture and had free access to water.

### 2.2. Induction of Anemia

For the induction of acute anemia, 40% of the blood volume was withdrawn according to the calculation of 8% of the body weight corresponding to the total blood volume [21]. The sheep were kept in left lateral decubitus position on a table with their limbs and necks restrained by assistants. Before induction of anemia, the animals had the right jugular region trichotomized and were aseptically treated with 10% povidone iodine.

Blood withdrawal was performed by jugular venipuncture with a previously described protocol [22]. Briefly, blood volume was withdrawn by gravity using an analytical scale. Each bag received 450 g of whole blood. The bags were sterile and contained anticoagulant Citrate Phosphate Dextrose Adenine (CPDA-1).

Animals were evaluated before induction (T0), 30 min (T30 min), 6 h (T6 h), 12 h (T12 h), and 24 h (T24 h) after blood loss. Clinical, hematological, biochemical, blood gas, lipid peroxidation, and oxidative stress variables were evaluated.

After 24 h of anemia induction, considering the best standards of animal welfare, all sheep were treated and received a transfusion of whole blood and were followed until full recovery, as described by Sousa et al. [15], who used the same animals from this study. A minimum number of sheep was used in this study since animals were divided in three groups of six sheep, following the study of Sousa et al. [15].

### 2.3. Clinical Evaluation

At the experimental times previously established, physical examinations of the animals were performed to check heart rate, respiratory rate, and rectal temperature. Physical exams were performed at the animal stall to avoid possible stress that could have affected the clinical measurement. Sheep were well adapted to the facilities and management.

For the blood count, 5 mL samples of blood were collected in vacuum tubes with anticoagulant ethylenediamine tetra-acetic acid (EDTA). The globular volume (GV), number of red blood cells, total hemoglobin, and number of leukocytes were determined on an automated hematological apparatus (BC Vet2000, Mindray, Shenzhen, China).

For blood gas evaluation, whole blood samples were collected through jugular venipuncture with disposable needles attached to 3 mL plastic syringes containing sodium heparin as an anticoagulant. Samples were stored in a thermal box, and the readings were taken in a hemogasometer (Cobas b 121, Roche^®^, Basel, Switzerland). Each determination was corrected according to the rectal temperature of the animal. Potential hydrogen (pH), partial pressure of carbon dioxide (pCO_2_), partial pressure of O_2_ (pO_2_), bicarbonate concentration (HCO_3_), and oxygen saturation (SO_2_) were evaluated.

### 2.4. Biochemical Evaluation

For the biochemical evaluation, blood samples were collected in vacuum tubes without anticoagulant, and were centrifuged at 1500× *g* for 15 min to obtain the serum. Creatinine, urea, total protein, and albumin concentrations were determined using automated biochemical analyzer (Randox, Crumlin, UK) [23]. Sodium and potassium concentrations were obtained in a hemogasometer (Roche).

### 2.5. Oxidative Stress and Lipid Peroxidation

To evaluate lipid peroxidation, the concentration of malondialdehyde (MDA) was analyzed, and to assess oxidative stress, the concentration of reduced Glutathione (GSH), Superoxide Dismutase (SOD), Glutathione Peroxidase (GPx), and Catalase (CAT) were analyzed.

Blood samples were collected in tubes containing lytic heparin as an anticoagulant for the analysis of SOD, GPx, and CAT. The samples were centrifuged at 2500 rpm for 10 min in a refrigerated centrifuge at 4 °C with slow deceleration, the plasma and the leukoplatelet layer (buffycoat) were removed, and subsequently, the RBCs were washed three times with phosphate solution (PBS 10%), until the supernatant was clear, and no leukocytes were present in the packed RBCs. The packed red blood cell obtained from the washes was stored in amber microtubes and frozen in a freezer at −40 °C for further SOD and GPx analysis. For the CAT analysis, 200 µL of the packed RBCs were removed and added to 1800 µL of the hemolyzer EDTA-β-mercaptoethanol. Then, a freeze–thaw cycle was performed at −20 °C for 10 min, followed by melting at room temperature for another 10 min, repeating the process for two consecutive times. The hemolysate obtained was stored at −40 °C in amber microtubes for further use.

Serum sample was used for measuring MDA [24] and GSH [25]. Meanwhile, serum SOD and GPx activities were determined on a Randox automatic biochemical analyzer (model RX Daytona, Crumlin, UK) using commercial Randox kits (RANSOD and RANSEL). Catalase activity was determined using its function of breaking down hydrogen peroxide into water and oxygen [26].

### 2.6. Statistical Analysis

Data were initially submitted to a Kolmogorov and Smirnov test, and since all variables presented normal distribution, they were submitted to analysis of variance using the PROC MIXED procedure (SAS 9.3, 2012) for repeated measures in time. In case of significant differences (*p* < 0.05), comparisons between times were performed using Tukey’s mean test.

## 3. Results

After blood loss, there was an increase in heart rate starting at time T30 min which remained until T24 h. The respiratory rate and temperature showed no changes after the baseline (Table 1).

With the withdrawal of blood at T0, there was a decrease in hematocrit values and number of red blood cells and hemoglobin from T30 on, with lower values observed at T24 h. The blood loss caused an increase in the number of leukocytes from T12 h on (Table 1).

Regarding the biochemical variables, after blood sampling, there was a reduction in total protein and albumin from T30 min, and these values remained constant until 24 h. Compared to the initial time, there was an increase in urea concentration from T6 h to T24 h (Table 1).

There was no variation in creatinine and sodium concentration after blood loss. The lactate levels were increased at T30, returning to the initial values at later moments. The potassium concentration decreased at T6 h, after which the values did not differ from baseline (Table 1).

Blood withdrawal promoted a decrease in blood pH and bicarbonate concentration at T30 min, with an increase in subsequent times (Table 2). There was an increase in the partial pressure of carbon dioxide at 24 h after blood loss, while the oxygen partial pressure decreased at 30 min and 6 h, returning to baseline values in the following moments. Oxygen saturation decreased at T30 min, with an increase in this variable at later times.

After blood loss, the activity of reduced glutathione showed lower values at T24 h, when compared to baseline. There were no changes in malonaldehyde concentration, nor in SOD, GPx, and catalase activity after blood loss (Table 2).

## 4. Discussion

The loss of blood volume can contribute to the emergence of multiple changes in the animals, triggering symptoms that can compromise metabolic and cellular functions. A reduction in blood volume decreases tissue oxygenation and cardiac output, and consequently, there is an increase in heart rate, which was observed from T30 min to T24 h [27,28,29].

The increase in HR occurs through the action of the sympathetic autonomic system in which nerve fibers are stimulated and activated by the drop in blood pressure and the consequent release of hormones such as vasopressin, angiotensin, and catecholamines [30]. The hormone angiotensin II, produced and secreted by the liver, affects blood pressure, since it acts as a vasoconstrictor in veins and arterioles, and together with the activity of the autonomic nervous system, increases the heart rate in an attempt to regulate blood pressure and blood volume [31].

The respiratory rate in this study did not change, even with the 40% loss in blood volume. It is believed that this is due to the compensatory mechanisms coming into play [31]. In goats, acute blood loss caused respiratory rate to increase four to five times above baseline after one hour of losing 15 and 30% of blood volume, respectively [5,8,32].

The body temperature of the sheep remained within normal values for the species (38.5–40 °C) at all times evaluated. Probably, physiological mechanisms of the central nervous system and peripheral centers were activated to regulate body temperature according to the homeostatic pattern. Thus, blood loss, combined with these different factors generated, was not sufficient for the onset of clinical hypothermia in animals. In goats, a two-degree increase in rectal temperature was observed one hour after blood loss, only returning to baseline values 24 days later [5].

The hematocrit, number of red blood cells, and hemoglobin concentration reduced from T30 min and reached lower values at 24 h after phlebotomy. Immediately after blood loss, the body initiates compensatory mechanisms in an attempt to maintain the circulating blood volume. The movement of fluid from the interstitial space into the intravascular medium under the action of osmotic pressure, as well as peripheral vasoconstriction and splenic contraction are activated in an attempt to maintain blood pressure and tissue oxygenation [31]. Thus, the hematological values in the first 12 h are not reliable because the compensatory mechanisms are activated, and the most reliable values are from T24 [5,6].

The leukocytes remained unchanged until T6 h, after which time there was an increase in the numbers of these cells above the reference values (4–12 × 10^3^/µL) [33]. Studies have reported a relationship between the increase in leukocyte cells and the hormones adrenaline and cortisol; although no measurements of glucocorticoids have been performed, they are believed to contribute to the development of leukocytosis [33,34]. The stress resulting from blood loss promotes the circulation of cytokines in the immune system, so that the actions performed interact with the central nervous system, resulting in the release of ACTH, which then acts in the secretion of cortisol into the bloodstream [35]. Another mechanism for the increase in leukocyte cells occurs through the mediation of norepinephrine in the autonomic nervous system; the defense cells of the immune system have receptors for this hormone, resulting in the release of cells into the bone marrow [35]. Previous studies on blood loss in sheep have shown the occurrence of leukocytosis [36,37], unlike what was observed in goats, where there are no numerical changes in these cells [5,8].

As for the blood gas parameters, the pH was reduced at T30 min, and increased in the following times. Peripheral circulation of non-vital organs may be reduced when there is a decrease in blood volume, leading the anaerobic metabolism to produce high amounts of lactate [31]. The excess of lactic acid induced a decrease in pH and a consequent decrease in bicarbonate concentration for buffering. After T30 min, the variables pH and bicarbonate increased; in addition, higher values of carbon dioxide pressure pCO2 were observed.

The partial pressure of oxygen and oxygen saturation showed a reduction at T30 min and T6 h, later returning to normal values. The reduction in these variables indicates a decrease in the amount of oxygen circulating in the body due to the loss of red blood cells [5,31].

After blood loss, total protein and albumin concentrations were reduced at T30 min, so that the values remained until T24 h. This fact occurred due to blood loss, which has several proteins in its constitution that regulate the plasma osmotic pressure [38].

Urea concentration can be affected by low tissue perfusion, resulting from hypovolemia. In these cases, renal blood flow decreases, promoting the reabsorption of urea in the renal tubules and the consequent increase in blood concentration [5,38]. Given this information, it is understood that the elevation in urea concentration resulted in a state of prerenal azotemia, with no changes in creatinine concentration.

Sodium concentration did not change after blood loss, but at T6 h, there was a reduction in potassium concentration, returning to basal values at the following moments. After losing 30% or 40% of blood volume, goats showed a reduction in the K concentration [7]. Sheep submitted to blood loss showed a reduction in blood sodium and potassium concentration probably due to the hemodilution resulting from blood loss [39].

The organism produces metabolites constantly, as a consequence of recurring cellular biochemical reactions; however, this mass production can collaborate with cellular toxicity in situations of low tissue oxygenation, and there is a need for the activation of means that contribute to the organism’s defense. The 40% loss in blood volume did not cause changes in the activity of the enzymes SOD, GPx, and catalase. The activity of these enzymes is associated with the control of the number of free radicals; thus, the erythrocyte antioxidant defense system was efficient in eliminating possible free radicals generated by blood loss [10,40,41].

A study with chronic blood loss from *Haemonchus contortus* parasitism in sheep showed reduced GPx activity and increased malonaldehyde [42]. In this study, no effect of lipid peroxidation was detected, since the concentrations of malondialdehyde (MDA) did not change, corroborating previous findings [37] that failed to detect changes in this variable after acute blood loss in sheep. Furthermore, blood loss led to a considerable MDA increase in rats, as previously reported [11].

GSH concentration was reduced at T24 h. GSH is a highly soluble antioxidant substrate that acts as an enzymatic cofactor for GPx to break hydrogen peroxide into oxygen and water. The reduced activity of this substrate can cause changes on the GPx enzyme in the performance of its converting capacity [43], which was not observed in this study, possibly because the animals were evaluated for only 24 h after blood loss. Animals naturally infected with *Haemonchus contortus* had reduced GSH and GPx concentrations, and the level of oxidative stress was related to parasite load [44].

Acute blood loss in this study caused oxidative stress; however, a recent report showed that sheep submitted to acute hemorrhage had reduced GSH values 24 h after blood loss and that transfusion of fresh or stored whole blood promoted the restoration of oxygen supply, and consequent return of initial GSH values [15]. Blood loss can occur due to different factors and different intensities. Acute blood loss is more difficult to examine due to the immediate necessity to treat animals. Here, we provide useful clinical information using a safe protocol that respected animal welfare.

## 5. Conclusions

The acute loss of 40% of blood volume promoted significant changes on hematological parameters, in addition to reduced oxygen pressure and development of temporary acidosis, with consequent activation of compensatory mechanisms, such as increased heart rate and reduced bicarbonate concentration.

Blood loss also contributed to the onset of oxidative stress with reduced GSH concentration, suggesting that this process generated enough free radicals to decrease the action of antioxidant substances.

## Figures and Tables

**Table 1:** Clinical, hematological, and biochemical variables of sheep following acute blood loss.

Variables	T0	T30 min	T6 h	T12 h	T24 h
HR (beats/min)	79.2 ± 11.9 ^b^	138.3 ± 34.0 ^a^	131.3 ± 25.1 ^a^	124.8 ± 26.0 ^a^	116.7 ± 23.2 ^a^
FR (mov/min)	36.8 ± 20.4	52.6 ± 37.9	35.2 ± 23.2	33.5 ± 27.9	33.7 ± 23.2
T (°C)	38.5 ± 0.5	38.4 ± 0.6	38.3 ± 0.7	38.4 ± 0.6	38.3 ± 0.5
Hematocrit (%)	34.4 ± 4.8 ^a^	27.6 ± 5.7 ^b^	24.6 ± 6.8 ^bc^	20.8 ± 5.5 ^cd^	17.4 ± 4.1 ^d^
RBCs (×10^6^)	9.5 ± 1.6 ^a^	7.5 ± 1.6 ^b^	6.7 ± 1.6 ^bc^	5.7 ± 1.4 ^cd^	4.8 ± 1.2 ^d^
Hemoglobin (g/dL)	11.3 ± 1.9 ^a^	8.9 ± 2.0 ^b^	7.9 ± 2.1 ^bc^	6.9 ± 1.9 ^cd^	5.6 ± 1.5 ^d^
Leukocytes (×10^3^)	5.4 ± 1.5 ^b^	4.7 ± 1.8 ^b^	8.8 ± 3.4 ^b^	26.6 ± 13.1 ^a^	26.5 ± 13.1 ^a^
Total Protein (g/dL)	7.1 ± 0.8 ^a^	5.1 ± 0.7 ^b^	5.2 ± 0.6 ^b^	5.3 ± 0.6 ^b^	5.1 ± 1.0 ^b^
Albumin (g/dL)	3.1 ± 0.3 ^a^	2.4 ± 0.2 ^b^	2.6 ± 0.1 ^b^	2.6 ± 0.1 ^b^	2.6 ± 0.1 ^b^
Urea (mg/dL)	30.7 ± 5.6 ^b^	52.9 ± 32.9 ^ab^	59.5 ± 33.9 ^a^	64.8 ± 32.1 ^a^	71.9 ± 37.3 ^a^
Creatinine (mg/dL)	1.1 ± 0.3	1.5 ± 0.9	1.5 ± 1.0	1.5 ± 0.9	1.4 ± 0.7
Lactate (mg/dL)	21.6 ± 10.4 ^b^	108.5 ± 52.3 ^a^	40.0 ± 28.3 ^b^	30.8 ± 16.7 ^b^	17.4 ± 7.9 ^b^
Sodium (mmol/L)	138.7 ± 3.3	141.5 ± 5.9	140.6 ± 6.0	143.0 ± 10.7	141.2 ± 6.3
Potassium (mmol/L)	4.4 ± 0.5 ^a^	4.0 ± 0.8 ^ab^	3.6 ± 0.6 ^b^	3.8 ± 0.7 ^ab^	3.9 ± 0.5 ^ab^

Different letters in the same row indicate statistical difference between time points (*p* < 0.05).

**Table 2:** Blood gas, lipid peroxidation, and oxidative stress variables of sheep following acute blood loss.

Variables	T0	T30 min	T6 h	T12 h	T24 h
pH	7.39 ± 0.06 ^b^	7.31 ± 0.08 ^c^	7.42 ± 0.05 ^ab^	7.45 ± 0.05 ^a^	7.44 ± 0.05 ^ab^
HCO_3_ (mmol/L)	21.3 ± 3.5 ^b^	16.8 ± 3.9 ^c^	25.2 ± 3.9 ^a^	25.4 ± 3.1 ^a^	26.0 ± 4.1 ^a^
pCO_2_ (mmHg)	35.1 ± 4.5 ^b^	35.3 ± 4.8 ^b^	39.1 ± 5.5 ^ab^	37.3 ± 3.7 ^ab^	39.8 ± 4.8 ^a^
pO_2_ (mmHg)	39.6 ± 8.9 ^a^	31.9 ± 5.7 ^b^	33.0 ± 6.7 ^b^	35.7 ± 5.3 ^ab^	34.7 ± 5.2 ^ab^
SO_2_ (%)	21.3 ± 3.5 ^b^	16.8 ± 3.9 ^c^	25.2 ± 3.9 ^a^	25.4 ± 3.1 ^a^	26.0 ± 4.1 ^a^
GSH (mg/dL)	25.7 ± 10.5 ^a^	21.6 ± 10.3 ^ab^	21.7 ± 9.6 ^ab^	18.7 ± 8.7 ^ab^	16.6 ± 7.9 ^b^
Malon (µmol/L)	0.49 ± 0.16	0.47 ± 0.17	0.39 ± 0.20	0.41 ± 0.21	0.45 ± 0.18
SOD (U/g Hg)	11.4 ± 3.6	12.2 ± 4.3	11.9 ± 3.5	12.3 ± 5.4	11.9 ± 3.8
GPx (U/g Hg)	3408.0 ± 1805.0	3928.5 ± 1804.9	3710.6 ± 1499.3	3599.1 ± 953.8	3986.7 ± 1613.9
Catalase (U/mg Hg)	2.67 ± 0.6	2.38 ± 0.7	2.25 ± 0.6	2.11 ± 0.7	2.58 ± 0.8

Different letters in the same row indicate statistical difference between time points (*p* < 0.05).

## Data Availability

The raw data are freely available upon request to the corresponding author.
